# IQM-PC332, a Novel DREAM Ligand with Antinociceptive Effect on Peripheral Nerve Injury-Induced Pain

**DOI:** 10.3390/ijms23042142

**Published:** 2022-02-15

**Authors:** Paula G. Socuéllamos, Luis A. Olivos-Oré, María Victoria Barahona, Pilar Cercós, Marta Pérez Pascual, Marina Arribas-Blázquez, José Ramón Naranjo, Carmen Valenzuela, Marta Gutiérrez-Rodríguez, Antonio R. Artalejo

**Affiliations:** 1 Department of Pharmacology and Toxicology, Veterinary Faculty and Instituto Universitario de Investigación en Neuroquímica (IUIN), Universidad Complutense de Madrid, 28040 Madrid, Spain; paulagarcia@iib.uam.es (P.G.S.); olivos@ucm.es (L.A.O.-O.); vbg@ucm.es (M.V.B.); martpe13@ucm.es (M.P.P.); marina.arribas@vet.ucm.es (M.A.-B.); 2 Instituto de Investigaciones Biomédicas Alberto Sols (IIBM), CSIC-UAM, 28029 Madrid, Spain; cvalenzuela@iib.uam.es; 3 Instituto de Investigación Sanitaria San Carlos, 28040 Madrid, Spain; 4 Instituto de Química Médica (IQM-CSIC), 28006 Madrid, Spain; pcercos@nebrija.es; 5 Centro Nacional de Biotecnología–Consejo Superior de Investigaciones Científicas (CSIC), 28049 Madrid, Spain; naranjo@cnb.csic.es; 6 Centro de Investigación Biomédica en Red Enfermedades Neurodegenerativas (CIBERNED), 28031 Madrid, Spain; 7 Centro de Investigación Biomédica en Red Enfermedades Cardiovasculares (CIBERCV), 28029 Madrid, Spain

**Keywords:** DREAM/KChIP3/calsenilin, DREAM ligands, neuropathic pain, nociception, chronic constriction nerve-injury, dorsal root ganglion neuron

## Abstract

Neuropathic pain is a form of chronic pain arising from damage of the neural cells that sense, transmit or process sensory information. Given its growing prevalence and common refractoriness to conventional analgesics, the development of new drugs with pain relief effects constitutes a prominent clinical need. In this respect, drugs that reduce activity of sensory neurons by modulating ion channels hold the promise to become effective analgesics. Here, we evaluated the mechanical antinociceptive effect of IQM-PC332, a novel ligand of the multifunctional protein downstream regulatory element antagonist modulator (DREAM) in rats subjected to chronic constriction injury of the sciatic nerve as a model of neuropathic pain. IQM-PC332 administered by intraplantar (0.01–10 µg) or intraperitoneal (0.02–1 µg/kg) injection reduced mechanical sensitivity by ≈100% of the maximum possible effect, with ED_50_ of 0.27 ± 0.05 µg and 0.09 ± 0.01 µg/kg, respectively. Perforated-patch whole-cell recordings in isolated dorsal root ganglion (DRG) neurons showed that IQM-PC332 (1 and 10 µM) reduced ionic currents through voltage-gated K^+^ channels responsible for A-type potassium currents, low, T-type, and high voltage-activated Ca^2+^ channels, and transient receptor potential vanilloid-1 (TRPV1) channels. Furthermore, IQM-PC332 (1 µM) reduced electrically evoked action potentials in DRG neurons from neuropathic animals. It is suggested that by modulating multiple DREAM–ion channel signaling complexes, IQM-PC332 may serve a lead compound of novel multimodal analgesics.

## 1. Introduction

Neuropathic pain is an unaddressed clinical problem due to its form of presentation (spontaneous pain, allodynia, and hyperalgesia), frequent chronicity, and the modest efficacy and dose-limiting effects of currently available drugs [1]. Neuropathic pain often involves a disfunction of primary nociceptive neurons having their soma in the dorsal root ganglions (DRG), which become hyperexcitable [2]. Abundant experimental evidence has linked changes in the expression and function of various ion channels (voltage-gated Na^+^, Na_v_, K^+^, K_v_, and Ca^2+^, Ca_v_, channels, transient receptor potential vanilloid-1, TRPV1 channel, etc_._) to hyperexcitability of DRG neurons in different models of neuropathic pain secondary to the injury of a peripheral nerve, thus making ion channels potential drug targets for the control of nociception [3]. In this context, an attractive strategy to achieve an effective and, above all, safe analgesic effect could be the development of drugs capable of interacting with the modulatory subunits of the channels rather than directly with their pore forming units.

The downstream regulatory element antagonist modulator (DREAM) is a multifunctional protein belonging to the family of neuronal Ca^2+^ sensors [4]. Initially identified as calsenilin, a presenilin binding protein [5], was rapidly recognized also as a transcriptional repressor through the association with DRE (Downstream Regulatory Element) sites [6], and a potassium channel interacting protein (KChIP3) that regulates the intracellular traffic and biophysical properties of K_v_4 channels [7]. Besides modifying the expression of several genes involved in pain perception, like c-fos [6], brain derived neurotrophic factor (BDNF) [8,9], prodynorphin [6,10], cathepsin L [11] or interleukins 2 and 4 [12], DREAM also interacts with a variety of ion channels and receptors involved in pain detection and transmission in DRG neurons. This is the case of NMDA glutamate receptors [13], TRPV1 channels [14], and a variety of voltage-activated channels, including K_v_4.3 channels responsible for the transient, A-type, potassium current (I_A_) [15], and the low, T-type [16], and high voltage-activated (HVA), L-type [17] Ca_v_ channels. Of note, except for NMDA receptors, DREAM acts to enhance currents through these channel types. Importantly, DREAM is expressed in DRG neurons [14,18,19], which would make it a suitable target for drugs potentially producing an analgesic effect by acting as DREAM ligands.

Here we investigated the mechanical antinociceptive effect of the a novel DREAM ligand IQM-PC332 (2-[2-(3,4-Dichlorophenyl)acetylamino]-4-(4′-n-butylphenyl)benzoic acid) in rats subjected to chronic constriction injury of the sciatic nerve (CCI), a common model of post-traumatic neuropathic pain. IQM-PC332 is known to bind with high affinity to DREAM (K_D_ = 0.28 µM in the surface plasmon resonance assay) and to inhibit currents through K_v_4.3/DREAM channels expressed in African green monkey kidney-derived CHO-K1 (CHO) cells with an IC_50_ = 6.8 µM [20,21]. Here, we show that IQM-PC332 exerts an antinociceptive effect upon local and systemic administration. Importantly, IQM-PC332 blocks I_A_ in rat DRG neurons with a similar potency and efficacy as it inhibits K_v_4.3/DREAM channels in CHO cells; likewise, IQM-PC332 reduces currents mediated by T-type and HVA Ca_v_ channels as well as TRPV1 channels in DRG neurons, all of which being actions that may underlie its analgesic effect. It is proposed that by modulating DREAM–ion channel interactions, small DREAM ligands may open new avenues for a “single-drug” multimodal analgesia.

## 2. Results

### 2.1. Effect of IQM-PC332 on Mechanical Sensitivity in CCI Animals

**Figure 1:**
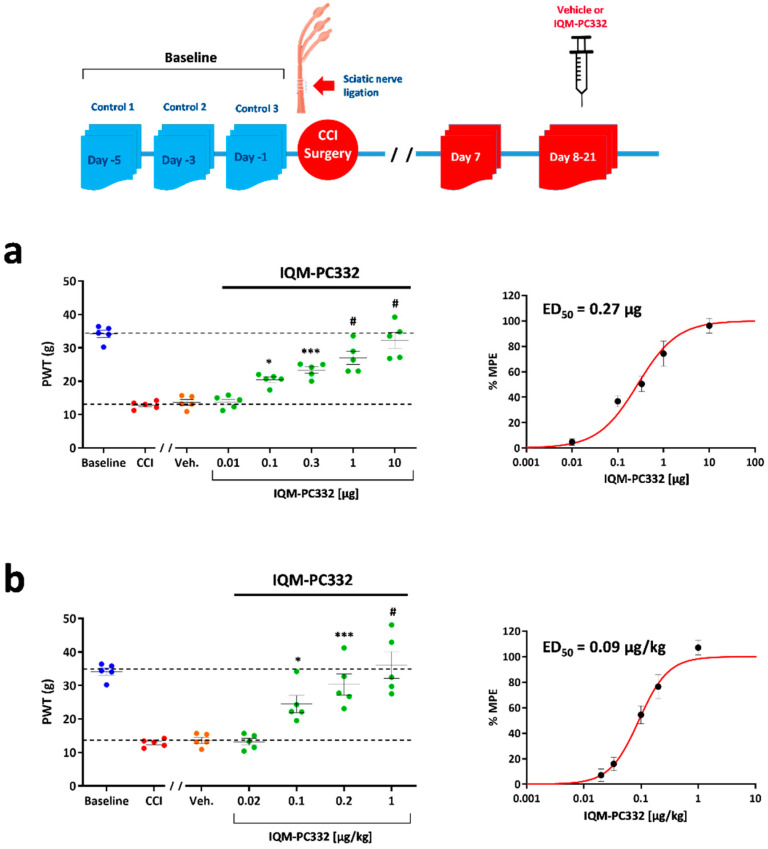
Effect of IQM-PC332 on mechanical sensitivity in CCI animals. A graphical scheme of the experimental design is depicted on top of the figure. IQM-PC332 or vehicle (DMSO, 3%) were administered by intraplantar (i.pl.) or intraperitoneal (i.p.) injection at 48 h intervals starting on day 7 after CCI surgery. Pre-CCI values (designated as Baseline) were taken as the mean of three determinations performed on days -5, -3, and -1 with respect to CCI surgery. (**a**) *Left panel*. Effect of IQM-PC332 on the paw withdrawal threshold (PWT) following i.pl. administration. *Right panel.* Dose–response curve of the effect of IQM-PC332 expressed as the percentage of the maximum possible effect (%MPE). (**b**) *Left panel*. Effect of IQM-PC332 on the paw withdrawal threshold (PWT) following i.p. administration. *Right panel.* Dose–response curve of the effect of IQM-PC332 expressed as the percentage maximum possible effect (%MPE). Dashed lines in left panels indicate PWT values at Baseline and 7 days after CCI. Data are given as the mean ± SEM of 5 measurements (*n* = 5 animals) for each administration route. Statistical significance was assessed by two-way ANOVA, followed by a Bonferroni’s post hoc test for comparisons at different doses with respect to vehicle. *: *p* < 0.05; ***: *p* < 0.001; #: *p* < 0.0001.

Mechanical sensitivity was evaluated before (baseline) and after CCI surgery, and was observed only in the CCI-injured hind paw. The paw withdrawal threshold (PWT) at baseline was 33.16 ± 0.98 g and was reduced to 12.83 ± 0.44 (*n* = 5) after CCI surgery, hence reflecting mechanical hypersensitivity (Figure 1). Importantly, the decrease in PWT to tactile stimulation remained stable from day 7 to day 21 post-CCI surgery so that the drug’s effects in vivo and in vitro could be studied during this time window.

The effect of IQM-PCC32 on mechanical sensitivity was assessed following intraplantar (i.pl.) and intraperitoneal (i.p.) administration. Intraplantar administration of IQM-PC332 (0.01–10 μg) in the affected hind paw increased PWT values while vehicle (dimethyl sulfoxide, DMSO, 3%) had no effect. PWT values were normalized by calculating the percentage of the maximum possible effect (%MPE), which peaked at ≈100% for the 10 µg dose (PWT of 32.28 ± 2.37; *n* = 5). Likewise, the ED_50_ for the effect of IQM-PC332 after i.pl. administration was 0.27 ± 0.05 µg (Figure 1a). Intraperitoneal administration of IQM-PC332 (0.02–1 µg/kg) also reduced mechanical sensitivity in the affected hind paw. %MPE was ≈100% at the 1 µg /kg dose (PWT of 36.13 ± 3.98; *n* = 5) and the ED_50_ was 0.09 ± 0.01 µg/Kg (Figure 1b). Interestingly, no effect of the drug on mechanical sensitivity could be observed in the contralateral, uninjured, hind paw (data not shown). Although this observation suggests that IQM-PC332 does not affect motor coordination, this issue was directly investigated with the RotaRod test. In Control, non-operated animals, IQM-PC332 (1 µg/kg, i.p.) did not affect the time to fall of the animals as compared to vehicle (data not shown). Altogether, these results indicate that IQM-PC332 attenuates mechanical hypersensitivity dose-dependently in the CCI model of neuropathic pain.

### 2.2. Effect of IQM-PC332 on I_A_ in DRG Neurons

**Figure 2:**
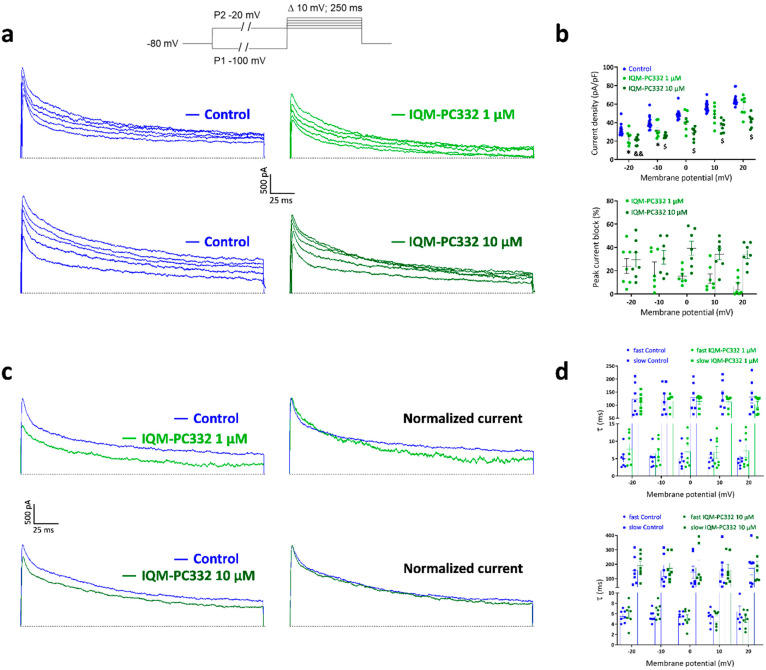
Effect of IQM-PC332 on peak amplitude and inactivation kinetics of I_A_ in DRG neurons from Control animals. (**a**) Representative recordings of I_A_ isolated by using the voltage protocol depicted on top of the panels. The effect of IQM-PC332 1 µM (*upper panel*) or 10 μM (*lower panel*) is shown. (**b**) *Upper panel*. Peak current density to voltage (I-V) relation in the absence (Control) and presence of IQM-PC332 1 µM and 10 μM, obtained from experiments as depicted in a; *lower panel*. Percent block of peak I_A_ by IQM-PC332 (1 μM and 10 μM) at different potentials. (**c**) Effect of IQM-PC332 1 μM (*upper panel*) and 10 μM (*lower panel*) on inactivation kinetics of I_A_. Currents evoked at 0 mV (left panels) from a were normalized to peak I_A_ in the absence of IQM-PC332 (right panels) to appreciate better the change in inactivation kinetics. (**d**) Effect of IQM-PC332 at 1 μM (upper graph) or 10 μM (lower graph) on time constants of inactivation at different potentials. The current records were fitted to a biexponential equation to obtain τ_f_ and τ_s_ values. Data are mean ± SEM from 7 cells (membrane capacitance of 29.92 ± 0.51 pF) for IQM-PC332 1 μM, and 7 cells (membrane capacitance of 34.54 ± 1.34 pF) for IQM-PC332 10 μM. Statistical significance in (**b**) was assessed by two-way ANOVA, followed by a Bonferroni’s post hoc test for multiple comparisons with respect to Control (membrane capacitance of 32.23 ± 0.94 pF; *n* = 14 cells). *: *p* < 0.05 for 1 µM; &&: *p* < 0.01, and $: *p* < 0.0001 for 10 µM).

IQM-PC332 has been characterized as an inhibitor of K_v_4.3/DREAM channels expressed in a heterologous system [20,21]. Since DRG neurons express DREAM as well as K_v_4.3 channels, which contribute to I_A_ [14,22,23], we first set out to record I_A_ in DRG neurons from unoperated, Control, animals to evaluate the effect of IQM-PC332 on native potassium channels. Figure 2a,c shows transient, fast activating and inactivating potassium currents in the voltage-range in which I_A_ makes a substantial contribution to K_v_ currents (from −20 mV to +20 mV). These currents were sensitive to 5 mM 4-aminopyridine (data not shown) and displayed biexponential inactivation kinetics (Figure 2a). Time constants of the fast (τ_f_) and slow (τ_s_) components at 0 mV were 5.26 ± 0.56 ms and 136.86 ± 22.48 ms, respectively, the fast component accounting for 28.03 ± 4.71% of the process (*n* = 14 cells). As previously reported [22], time constants exhibited weak voltage-dependence (Figure 2d). IQM-PC332 at 1 µM and 10 μM reduced the peak amplitude of I_A_ dose-dependently, the effect being particularly larger at 10 µM in the range from 0 to +20 mV (Figure 2a). This partly reflects the voltage dependence of the block at 1 µM, which decreases with the depolarization (percent block of 24.12 ± 8.97% and 6.82 ± 5.63 at −20 mV and +20 mV, respectively; *n* = 7 cells), and that would be consistent with a preferential binding of the drug to the close-activated state of the channel (Figure 2b). IQM-PC332 1 μM slightly accelerated current inactivation by reducing τ_s_, whereas at 10 µM it slowed the inactivation by increasing both τ_f_ and τ_s_ (Figure 2c,d). These results are in line with those reported for heterologously expressed K_v_4.3/DREAM channels and point to a similar effect of IQM-PC332 on native K_v_4.3/DREAM channels.

Next, we measured I_A_ in DRG neurons from CCI animals. In agreement with previous reports [24,25], there was a decrease of I_A_ in these neurons (Figure S1). At +20 mV, peak I_A_ in DRG neurons from CCI animals was 49.91 ± 2.14 pA/pF (*n* = 10 cells), which was significantly smaller than that recorded from Control animals (65.15 ± 1.77 pA/pF; *n* = 14 cells; *p* < 0.0001). IQM-PC332 (1 µM and 10 µM) also inhibited peak amplitude of I_A_ dose-dependently in DRG neurons from neuropathic animals. No significant difference in percent block was observed at −20 mV for 1 µM (33.15 ± 7.20%; *n* = 5 cells) and 10 µM (66.74 ± 13.36%; *n* = 5 cells) IQM-PC332 with regard to Control animals (23.98 ± 6.40% and 29.41 ± 6.29% for 1 and 10 µM, respectively; *n* = 7 cells for each drug concentration), thereby implying a similar action of the drug on I_A_ in DRG neurons from Control and CCI animals (Figure S1).

### 2.3. Effect of IQM-PC332 on T-Type and HVA Ca^2+^ Currents in DRG Neurons

Ca_v_ channels are known to play a key role in the control of excitability and neurotransmitter release in DRG neurons [26,27,28]. They act as “pronociceptive” channels [27,29,30] and, consequently, are potential drug targets for the treatment of acute and chronic pain [31,32].

**Figure 3:**
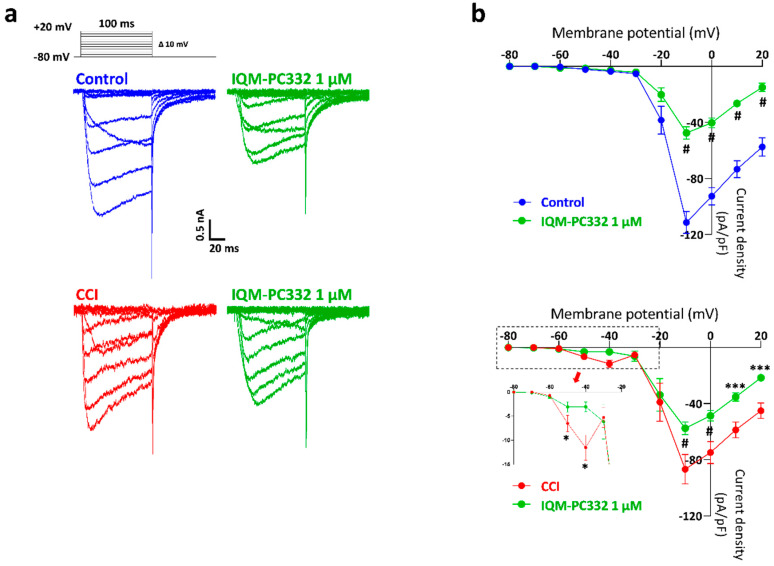
Effect of IQM-PC332 (1 µM) on T-type and HVA Ca^2+^ currents in DRG neurons from Control and CCI animals. (**a**) Representative recordings of T-type and HVA Ca^2+^ currents in DRG neurons from Control and CCI animals in the absence and presence of IQM-PC332 1 µM. Currents were evoked by voltage pulses as depicted in the inset. (**b**) Peak I-V curves obtained from current traces in Control (*upper panel*) and CCI (*lower panel*) DRG neurons in experiments as depicted in (**a**). The inset in the lower panel shows an expanded view of the I-V curve at negative potentials (from −80 mV to −20 mV). Data are mean ± SEM of 5 (membrane capacitance of 29.02 ± 0.92 pF) and 7 (membrane capacitance of 29.37 ± 1.14 pF) cells per point from Control and CCI animals, respectively. Statistical significance was assessed by two-way ANOVA, followed by a Bonferroni’s post hoc test for paired comparisons at different potentials with respect to Control or CCI. *: *p* < 0.05; ***: *p* < 0.001; #: *p* < 0.0001.

We characterized the effect of IQM-PC332 on T-type and HVA Ca^2+^ currents in DRG neurons from both Control and CCI animals. T-type and HVA Ca^2+^ currents differ in voltage for activation as well as in inactivation and deactivation kinetics [33]. T-type Ca^2+^ currents activate at low voltages, ≈−50 mV, reach maximal amplitude at −40 mV, undergo fast and pronounced inactivation, and show slow deactivation upon repolarization; at variance, HVA Ca^2+^ currents start to activate at ≈−30 mV, peak at −10 mV, and exhibit slow inactivation. We made use of a series of step-like depolarizations from a V_h_ = −80 mV up to +20 mV in 10 mV increments to record both T-type and HVA Ca^2+^ currents in DRG neurons (Figure 3). In cells from Control animals, small size T-type Ca^2+^ currents were observed in the voltage range between −50 and −30 mV (3.53 ± 0.99 pA/pF at −40 mV; *n* = 5 cells); in line with previous reports [34], DRG neurons from CCI animals exhibited larger T-type Ca^2+^ currents, with notorious transient kinetics on sustained depolarization (11.56 ± 2.60 pA/pF at −40 mV; *n* = 7 cells; *p* = 0.03) (Figure 3a). Accordingly, I-V curves exhibited a single peak corresponding to HVA Ca^2+^ currents in DRG neurons from Control animals (111.40 ± 7.84 pA/pF at −10 mV), while a prominent peak of HVA Ca^2+^ currents (86.73 ± 10.48 pA/pF at −10 mV; *p* > 0.05) preceded by a hump, the latter corresponding to T-type Ca^2+^ currents, was observed in DRG neurons from CCI animals (Figure 3b).

IQM-PC332 (1 µM) reduced Ca^2+^ currents at all voltages tested, thus suggesting an action on both T-type and HVA Ca_v_ channels (Figure 3a,b). It is of note that IQM-PC332 neither affected the kinetics of the current nor the position of the peak and the hump in I-V curves from Control and CCI animals (Figure 3a,b).

**Figure 4:**
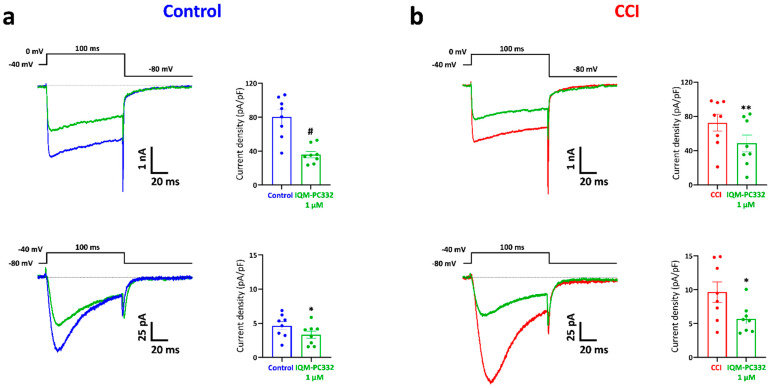
Effect of IQM-PC332 (1 µM) on isolated T-type and HVA Ca^2+^ currents in DRG neurons from Control and CCI animals. Representative recordings of HVA (upper) and T-type (lower) Ca^2+^ currents in DRG neurons from Control (**a**) and CCI (**b**) animals in the absence (blue or red traces) and presence of IQM-PC332 1 µM (green traces). T-type Ca^2+^ currents were elicited by a depolarization to −40 mV from a V_h_ of −80 mV, whereas HVA Ca^2+^ currents were activated at 0 mV from a V_h_ of −40 mV (see voltage protocols at the top of the recordings). The scatter graphs show current density values for each condition. Data are mean ± SEM from 8 cells for Control (25.96 ± 2.77 pF) and CCI (28.29 ± 3.03 pF) conditions. Statistical significances were assessed by using a Student’s *t*-test for paired comparisons. *: *p* < 0.05; **: *p* < 0.01; #: *p* < 0.0001.

To better determine the effect of IQM-PC332 (1 µM) on T-type and HVA Ca^2+^ currents, we applied two different pulse protocols to record independently T-type and HVA Ca^2+^ currents from the same cell [34] (Figure 4). T-type Ca^2+^ currents were elicited by a depolarization to −40 mV from a V_h_ of −80 mV, whereas HVA Ca^2+^ currents were activated at 0 mV from a V_h_ of −40 mV, which fully inactivates T-type Ca^2+^ currents. T-type and HVA Ca^2+^ currents hence recorded differed both in amplitude and kinetics in Control conditions, the former having a current density of 4.65 ± 0.60 pA/pF (*n* = 8 cells) and a marked inactivation, while the latter inactivated barely but exhibited a much larger current density of 80.15 ± 8.50 pA/pF (Figure 4a). By using these protocols, we confirmed the enlargement of T-type Ca^2+^ currents in neurons from CCI animals (9.64 ± 1.50 pA/pF; *n* = 8 cells; *p* = 0.008) whereas HVA Ca^2+^ currents were mildly reduced as compared to Control conditions (72.62 ± 9.78 pA/pF; *p* = 0.57) (Figure 4b). IQM-PC332 (1 µM) blocked both T-type and HVA Ca^2+^ currents in a statistically significant manner. Interestingly, percent block of HVA Ca^2+^ currents was larger in cells from Control (53.87 ± 3.24%) than from CCI (36.54 ± 7.71%; *p* = 0.049) animals; inhibition of T-type Ca^2+^ currents by IQM-PC332 was similar in CCI cells (30.71 ± 9.95%) and Control ones (28.19 ± 6.12%; *p* = 0.83).

### 2.4. Effect of IQM-PC332 on TRPV1 Channels in DRG Neurons

TRPV1 is a non-selective cation channel localized in peripheral sensory neurons where it activates in response to a variety of stimuli (heat, low pH, endocannabinoids), but it can also be activated by natural compounds (i.e., capsaicin, resiniferatoxin, etc.) [35]. TRPV1 activation by capsaicin induces a nociceptive sensory neuron depolarization that translates into transient pain sensations. Interestingly, mechanical hypersensitivity in CCI animals is reversed by i.pl. administration of capsazepine (30 µg), a TRPV1 channel blocker, thus suggesting the involvement of TRPV1 receptors in mechanical hypersensitivity (Figure S2).

**Figure 5:**
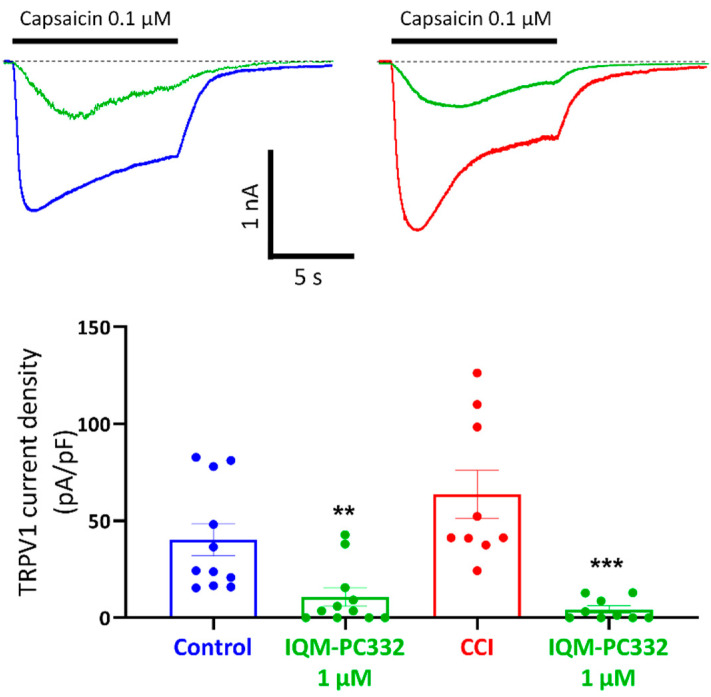
Effect of IQM-PC332 (1 µM) on capsaicin-evoked currents in DRG neurons from Control and CCI animals. *Upper panels*. Representative recordings of 0.1 µM capsaicin-evoked currents in the absence (blue or red) and the presence of IQM-PC332 (green) in DRG neurons from Control (*upper left panel*) and CCI (*upper right panel*) animals. Horizontal bars on top of the recordings indicate the time of capsaicin application. *Lower panel*. Scatter plot of peak current densities evoked by capsaicin in the absence and the presence of IQM-PC332 (1 μM). Data are means ± SEM from 11 (membrane capacitance of 28.46 ± 2.44 pF) and 9 (membrane capacitance of 31.93 ± 3.65 pF) cells of Control and CCI animals, respectively. Statistical significance was assessed by the Student’s *t*-test for unpaired comparisons. **, *p* < 0.01; ***, *p* < 0.001 with respect to capsaicin alone.

We therefore investigated the ability of IQM-PC332 to modify the responses evoked by capsaicin in DRG neurons. Figure 5 shows that IQM-PC332 (1 µM) markedly inhibited peak capsaicin (0.1 µM) currents in cells from Control (40.32 ± 8.34 pA/pF in the absence, and 10.78 ± 4.66 pA/pF in the presence of IQM-PC332; *n* = 11 cells; *p* < 0.01) and CCI (63.63 ± 12.44 pA/pF in the absence, and 4.32 ± 1.86 pA/pF in the presence of IQM-PC332; *n* = 9 cells; *p* < 0.001) animals. Interestingly, TRPV1-mediated currents were upregulated in CCI animals (*p* = 0.043) and percent block by IQM-PC332 was also larger in neurons from this type of animals (93.20% in CCI animals versus 73.52% in Control ones).

### 2.5. Effect of IQM-PC332 on Electrical Activity of DRG Neurons

**Figure 6:**
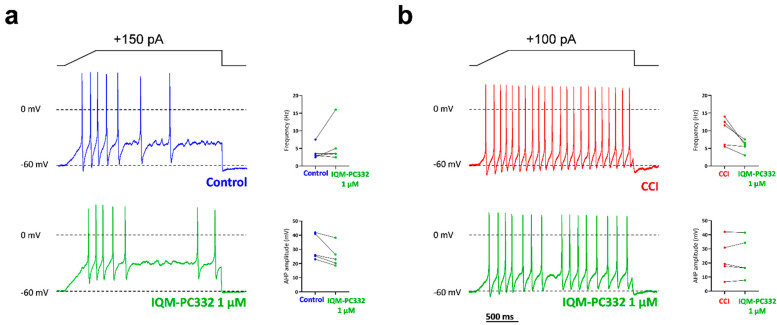
Effect of IQM-PC332 on electrical excitability in DRG neurons from Control and CCI animals. (**a**) Current-clamp recordings of action potentials evoked by current injection in DRG neurons from Control animals in the absence (blue) and the presence (green) of IQM-PC332 (1 µM). (**b**) Current-clamp recordings of action potentials evoked by current injection in DRG neurons from CCI animals in the absence (blue) and the presence (green) of IQM-PC332 (1 µM). Current protocols are shown at the top of the panels. Insets show before–after plots of action potential frequency and the amplitude of the action potential afterhyperpolarization (AHP). V_comm_ = −60 mV. Recordings are representative of those obtained in 5 cells from Control and CCI animals.

Last, we investigated the effect of IQM-PC332 (1 µM) on electrical activity evoked by current injection in DRG neurons from both Control and CCI animals (Figure 6). Current injection evoked the firing of action potentials at higher frequency in neurons from CCI animals (9.90 ± 1.74 Hz; *n* = 5 cells) than from Control ones (3.90 ± 0.91 Hz; *n* = 5 cells; *p* = 0.0094), which suggests increased cellular excitability in nociceptive neurons from neuropathic animals as previously reported [25,36]. Interestingly, IQM-PC332 (1 µM) did not affect the frequency of firing of action potentials in 3 out of 5 cells tested from Control animals, despite the fact that it consistently diminished the amplitude of action potential afterhyperpolarizations (AHP), in agreement with the marked inhibition of HVA Ca^2+^ currents observed in these animals (Figure 6a). In contrast, IQM-PC332 (1 µM) reduced the rate of action potential firing in 5 out of 5 cells from CCI animals. Of note, only a small reduction in AHP amplitude was observed in 2 cells, which is in accord with the lower effect of IQM-PC332 on HVA Ca^2+^ channels in neurons from CCI animals. This result points to the involvement of other channels in the effect of IQM-PC332 in electrical activity in DRG neurons from neuropathic animals (Figure 6b).

## 3. Discussion

We evaluated the effect of IQM-PC332, a novel DREAM ligand, on nociceptive hypersensitivity secondary to the injury of the rat’s sciatic nerve, and on different ionic conductances and electrical excitability in primary nociceptive DRG neurons from Control and CCI animals. The main results show that IQM-PC332 reduced mechanical hypersensitivity in vivo and inhibited I_A_, Ca_v_ and TRPV1 currents as well as action potential firing in isolated neurons from CCI animals. It is therefore proposed that IQM-PC332 acts as a multimodal ion channel modulator to produce analgesia in animals with chronic nociceptive hypersensitivity.

IQM-PC332 alleviated mechanical hypersensitivity following acute i.pl. and i.p. administration. We tested mechanical sensitivity at the central part of the plantar surface of the hind paw, which in animals subjected to sciatic nerve injury reflects both the response of fibers of the tibial branch of the sciatic nerve and also of fibers of the saphenous nerve, that reinnervate the skin of the lesioned hind paw [37,38,39] The effect of the drug was clearly separated from that of the vehicle, was not confounded by a modification of motor coordination, and was not observed in Control, unoperated animals, hence excluding an action on normal nociception. Moreover, the antinociceptive effect was dose-dependent, reaching a maximum effect of ≈100%. The fact that the effect is achieved by both local and systemic administration suggests a peripheral site of action, likely on DRG neurons, the primary nociceptive neurons in the pain pathway.

Nociceptive DRG neurons transduce noxious stimuli causing pain, thereby functioning as nociceptors, and generate action potentials that propagate to the spinal cord where nociceptive information is transmitted to the second-order sensory neurons, which will process and convey it to upper levels of the nervous system. Multiple ion channels are involved in sensing and transmitting nociceptive information in DRG neurons, including polymodal TRPV1 channels activated by a diversity of stimuli (thermal, mechanical and chemical) [35,40], Na_v_ and K_v_ channels, responsible for action potential generation [23,41], and Ca_v_ channels, participating in both neurotransmitter release at central terminals and the regulation of action potential firing at the cell soma [31,32,42,43,44]. IQM-PC332 has been previously characterized as a potent ligand of DREAM that inhibits currents mediated by DREAM/K_v_4.3 complexes in CHO cells with an IC_50_ of 6.8 µM [20]. DRG neurons are known to express both DREAM (KChIP3) and K_v_4.3 channels, which contribute to I_A_ [14,18,22,25]. Our results indicate that IQM-PC332 modulates I_A_ in DRG neurons, hence suggesting that it acts on native DREAM/K_v_4.3 complexes at the plasma membrane. Importantly, IQM-PC332 dose-dependently (1 and 10 µM) inhibited peak I_A_, and produced a small delay in current inactivation at 10 µM, both effects being consistent with those reported in CHO cells expressing DREAM/K_v_4.3 complexes. Likewise, I_A_ inhibition at 1 µM IQM-PC332 appears to be voltage-dependent, diminishing with the depolarization, which in accordance with previous data suggests that the drug interacts with the close-activated state of the channel [45]. This implies that the effect of IQM-PC332 could wane during the peak of action potential, and thus have little impact on action potential repolarization.

We also investigated the effect of IQM-PC332 on other ionic conductances known to be modulated by DREAM, and which are also potential targets for analgesic drugs. IQM-PC332, also at concentrations at which it interacts with DREAM, reduced currents through T-type and HVA Ca_v_ channels. The effect spans the voltage-range of activation of the two channel classes, so that a broad effect on DRG neuron function can be achieved. This effect would result mainly in the inhibition of neurotransmitter release at the central terminals of DRG neurons, which is controlled by presynaptic N- and P/Q-type, HVA Ca_v_ channels [46,47], and in a reduction in action potential firing brought about by inhibition of T-type Ca_v_ channels. Importantly, T-type Ca_v_ channels are upregulated following CCI and contribute to the increased electrical excitability and glutamate release in DRG neurons of neuropathic animals (present results; [34,48]). By acting predominantly on hyperexcitable neurons, IQM-PC332 may exert a selective antinociceptive effect, and thus preserve normal nociception. This notion was substantiated by recording action potentials evoked by current injection in DRG neurons from Control and CCI animals. In Control cells, IQM-PC332 barely affected or even increase the rate of action potential firing. This effect could possibly be attributed to blockade of HVA Ca_v_ channels with the ensued reduction in the amplitude and duration of the AHP [49], as well as to the inhibition of I_A_, which would shorten the interval between consecutive action potentials [50]. In contrast, in neurons from CCI animals, IQM-PC332 reduced the rate of firing as expected from a dominant effect on T-type Ca_v_ channels that would counteract the possible effect on I_A_ and HVA Ca^2+^ currents, which are reduced in cells from neuropathic animals (present results; [25,49]).

Interestingly, CCI animals develop a TRPV1-related algesic tone as deduced from the ability of local capsazepine to alleviate mechanical hypersensitivity in the injured hind paw. This result points to TRPV1 channels as a therapeutic target in the CCI model of neuropathic pain. Importantly, IQM-PC332 (1 µM) reduced TRPV1 channel currents, hence providing another possible mechanism of action for its analgesic effect.

In sum, the effects of IQM-PC332 on ion conductances in DRG neurons here reported would affect: (i) sensory transduction through inhibition of TRPV1 channels; (ii) frequency coding of information by decreasing I_A_ and T-type Ca_v_ channels, and (iii) synaptic transmission at the spinal cord by inhibiting HVA Ca_v_ channels. The effects on TRPV1 channels and on Ca_v_ channels will depress DRG neuron function and are likely related to the behavioral effect of IQM-PC332. Importantly, upregulation of TRPV1 and T-type Ca_v_ channels in DRG neurons, and the appearance of a capsazepine-sensitive tone in neuropathic animals [34,51], possibly explain why IQM-PC332 alleviates mechanical hypersensitivity without altering normal mechanical nociception.

IQM-PC332 thus acts as a multimodal ion channel modulator in DRG neurons. This would relate to its ability to perturb DREAM–ion channel interactions at the plasma membrane. IQM-PC332 modulation of I_A_ in DRG neurons is consistent with previous data indicating that IQM-PC332 binds to DREAM and silences DREAM’s effect on Kv4.3 channels expressed in CHO cells [20,21]. Likewise, the effect of IQM-PC332 on HVA Ca_v_ channels could be related to the fact that DREAM (KChIP3) coimmunoprecipitates with HVA Ca_v_1.2 (L-type) channels and that IQM-PC332 blocks (IC_50_ of 32.64 nM) such a DREAM–Ca_v_ channel interaction (J.R.N, personal communication). Interestingly, KChIP2, another member of the KChIP family, increases ion flux through cardiac L-type channels [17], which would give rationale to the inhibition of HVA Ca_v_ currents by IQM-PC332 in sensory neurons. Furthermore, DREAM interacts with TRPV1 channels to increase TRPV1 currents in DRG neurons [14], making sense of the inhibition of capsaicin-activated currents by IQM-PC332. Lastly, DREAM mediates the interaction between T-type Ca_v_ channels and K_v_4.3 channels, so that the latter increases their availability to open in a Ca^2+^-dependent manner. It seems therefore plausible that by binding to DREAM, IQM-PC332 could inhibit T-type Ca_v_ channels directly or indirectly through K_v_4.3 channels [16,52]. IQM-PC332 has been proposed to interact with multiple residues of DREAM (Leu96, Phe100, Ile117, Tyr118, Phe121, Tyr130, Phe151, Leu155, Leu158, Leu159, Ile194 and Ile256), which are mostly located in a large hydrophobic cavity surrounded by the N- and C-terminus of the protein [20,21]. Interestingly, both the N- [13,14] and C-terminus [52] of DREAM interact with ion channels, hence making it possible for IQM-PC332 to affect those interactions.

Although modulation of ion channels at the plasma membrane is the most likely mechanism underlying the analgesic effect of IQM-PC332, we cannot rule out an effect on gene expression. This is because behavioral evaluation was performed 20 min after drug administration, and IQM-PC332 has been shown to interfere with DREAM-mediated transcriptional repression of c-fos expression in STHdhQ^7/7^ neuroblastoma cells in just 15 min after drug exposure [20]. Since DREAM controls prodynorphin and BDNF gene expression and both DREAM knock-out and its constitutive activation have been reported to affect nociceptive processing [9,10,53], it is also possible that changes in gene expression could be involved in the antinociceptive effect of IQM-PC332.

In conclusion, IQM-PC332 could serve as lead compound for the development of novel DREAM ligands useful for a multimodal treatment of neuropathic pain by virtue of a concerted action on a variety of DREAM-interacting signaling complexes. Further studies for a clear separation of genomic and non-genomic effects of DREAM ligands will certainly lead to a better elucidation of the role of DREAM in nociception and of the therapeutic utility of small-drug DREAM ligands.

## 4. Materials and Methods

Adult male Sprague Dawley rats (weighing 200–220 g/6–8 weeks old) were used in the experiments. Animals were housed in transparent cages with temperature controlled at 23 °C in a 12 h light/dark cycle room; water and food were provided ad libitum. All experimental procedures were conducted according to the animal welfare guidelines of the European Community (European Directive 2010/63/UE) to minimize animal suffering and were approved by Universidad Complutense de Madrid and Comunidad de Madrid Committee on Animal Experimentation (PROEX 207.8/21).

### 4.1. Chronic Constriction Injury of The Sciatic Nerve

CCI was performed according to Bennett and Xie (1988) [54]. Briefly, rats were anesthetized with i.p. ketamine (100 mg/kg; Merial Labs, Barcelona, Spain) and medetomidine (100 µg/kg; Esteve Labs, Barcelona, Spain). Under sterile conditions, approximately 7 mm of the right nerve was freed proximal to the sciatic trifurcation, and four barely constricting ligatures (1 mm apart) using 4/0 chromic catgut were applied. The incision was closed in layers with silk thread 6/0. Animals were then allowed to recover from surgery for 7 days before being used in additional procedures.

### 4.2. Behavioural Testing

#### 4.2.1. Mechanical Sensitivity

Rats were habituated to the experimental setting for at least 30 min before testing. All tests were conducted between 09:00 and 12:00. Mechanical sensitivity was evaluated with a dynamic plantar aesthesiometer (Ugo Basile, Gemonio, Italy) by means of a 0.5 mm filament exerting increasing force (up to 50 g over 20 s) onto the central area of the plantar surface of the hind paw until the animal lifted its paw, the actual force at that time was automatically registered (paw withdrawal threshold; PWT). Hypersensitivity was defined as at least a 25% decrease in PWT compared with values before CCI surgery. PWT measurements were repeated 3 times at 5 min intervals, and the mean value was reported.

PWT determination was carried out before surgery (mean of 3 measurements on alternate days the week preceding surgery, collectively designated as Baseline) and on post-surgery days 7–21, when abnormal nociceptive behaviour was at a stable maximum. Neuropathic rats were allocated into 2 groups to receive IQM-PC322 and vehicle (DMSO 3%) by either the i.pl. (20 µL; *n* = 5 animals) or i.p. (250 µL; *n* = 5 animals) route. Responses to mechanical stimulation were assessed 20 min before and 20 min after IQM-PC332 or vehicle injection. Each animal received six injections with a separation of 48 h.

PWT values were transformed into percentage of the maximum possible effect (%MPE), and %MPE was calculated as follows: (Drug PWT-Vehicle PWT)/Baseline PWT)-Vehicle PWT) × 100. Dose-response curves and ED_50_ values were obtained by nonlinear regression with variable slope using version 8 (GraphPad Software, Inc., San Diego, CA, USA).

#### 4.2.2. Motor Coordination

Motor coordination was assessed with a RotaRod apparatus (Ugo Basile, Gemonio, Italy). Non-operated rats were trained in the experimental procedure for at least two days. Motor coordination was evaluated through the time the animal spent on a roller rotating at a continuous speed (16 rpm). The cut-off time of 90 s was divided into two periods of 45 s, and the times spent in each period were summed [55]. After obtaining control values, the animals were evaluated 30 min after i.p. administration of IQM-PC332 (1 µg/kg) or vehicle treatment (DMSO 3%).

### 4.3. Isolation of DRG Neurons

Rats were sacrificed by cervical dislocation followed by decapitation and lumbar segments of the spinal column were removed and placed in a cold Ca^2+^, Mg^2+^-free Hank’s solution (Sigma-Aldrich, Madrid, Spain). The bone surrounding the spinal cord was removed and the right L4, L5 and L6 DRG were exposed and pulled out. After removing the roots, DRG were chopped in half and incubated for 60 min at 37 °C in Dulbecco’s modified Eagle’s Medium-low glucose (DMEM; Sigma-Aldrich) containing 5 mg/mL collagenase XI (Sigma-Aldrich, Madrid, Spain), 100 U/mL penicillin (Sigma-Aldrich), and 0.1 mg/mL streptomycin (Sigma-Aldrich). Then, the cell suspension was washed with DMEM by centrifugation (300 G, 5 min at 4 °C), filtered through a 100 µm mesh to eliminate cell clumps and washed again by centrifugation. The cell pellet was resuspended in DMEM and 40 µL was dropped onto 10 mm diameter glass coverslips treated with poly-d-lysine (1 mg/mL, 30 min; Sigma-Aldrich) placed in 35 mm diameter Petri dishes. Finally, plated cells were flooded with 2.5 mL of DMEM supplemented with 10% fetal calf serum (Sigma-Aldrich, Madrid, Spain), 100 U/mL penicillin and 0.1 mg/mL streptomycin, and stored in an incubator (Hera Cell, Heraeus, Hanau, Germany) in a 5% CO_2_ atmosphere at 37 °C. This protocol yields spherical cell bodies without neurites, from which only small to medium DRG neurons (diameter <30 μm) [23,36] were chosen for recording within 12–24 h of plating.

### 4.4. Electrophysiological Recordings

All electrophysiological recordings were performed in the perforated-patch variant of the whole-cell configuration of the patch–clamp technique with an EPC10/2 amplifier using PatchMaster software (HEKA Electronic, Lambrecht, Germany) [56]. Patch pipettes were made from borosilicate glass and fire-polished to a resistance of 4.5–5.5 MΩ when filled with an internal solution. Membrane currents were filtered at 3 kHz, and sampled at 10 kHz from cells held at a voltage (V_h_) of −80 mV. Series resistance (<20 MΩ) was compensated by 80% and monitored throughout the experiment together with the cell membrane capacitance. Cells in which series resistance changed by more than 20% or holding current exceeded 20 pA were discarded. Membrane potentials were recorded under current-clamp conditions and filtered at 3 kHz. Action potentials were evoked by current injection in DRG neurons from a V_comm_ of −60 mV. Experiments were performed at room temperature (22–25 °C).

#### 4.4.1. Solutions and Drug Application

The standard extracellular solution contained (mM) 145 NaCl, 2.8 KCl, 3 CaCl_2_, 1 MgCl_2_, 10 4-(2-Hydroxyethyl)piperazine-1-ethanesulfonic acid, N-(2-Hydroxyethyl) piperazine-N′-(2-ethanesulfonic acid) (HEPES), and 12 glucose (pH 7.35 adjusted with NaOH; ≈320 mOsm) that was constantly superfused at a rate of approximately 1 mL × min^−1^. To eliminate currents through Na_v_ channels during recordings of voltage-gated Ca^2+^ currents, the standard solution was modified by replacing NaCl with N-Methyl-d-glucamine, the pH being adjusted with HCl.

The internal solution used to record K_v_ currents contained (mM) 145 KCl, 2 MgCl_2_, 0.3 EGTA, 0.3 GTP.Li_3_, 2 ATP.Na_2_, 10 HEPES (pH 7.2 adjusted with KOH; ≈310 mOsm). In experiments aiming to isolate Ca_v_ currents, the internal solution contained (mM): 140 CsCl, 2 MgCl_2_, 10 EGTA, 0.3 GTP.Li_3_, 2 ATP.Na_2_, 10 HEPES (pH 7.2 with CsOH; ≈307 mOsm). The perforated-patch configuration was obtained using amphotericin B (Sigma-Aldrich). Amphotericin B was dissolved in dimethyl sulfoxide (DMSO) and stored at −20 °C in aliquots of 50 mg/mL. To seal the cells more easily, the patch pipette was immersed for a few seconds into an internal solution without amphotericin B and then back-filled with the internal solution containing amphotericin B (50–100 µg/mL). After sealing, series resistance decreased gradually to reach values below 20 MΩ within 10 min. Fresh pipette solution containing amphotericin was prepared every 2 h.

IQM-PC332, synthesized following protocols previoulsy described [20], was dissolved in DMSO at a stock concentration of 1 mM, and added to the external solution at the desired concentration in each experiment. The drug was directly applied for 2–4 min onto the cell under investigation by means of a multibarrel concentration-clamp device coupled to electronically driven miniature solenoid valves under the control of PatchMaster software. Capsaicin (Sigma-Aldrich) was dissolved in the extracellular solution to a concentration of 0.1 µM, and was applied with a glass-pipette (3–5 μm tip diameter) placed near (5–10 μm) the cell of interest with a pneumatic drug ejection system (PDES-02DX, NPI Electronic GmbH, Germany). Capasicin experiments in the absence and the presence of IQM-PC332 were conducted in separated cells to avoid TRPV1 channel desensitization upon repeated stimulation.

#### 4.4.2. Voltage/Current Protocols and Data Analysis

Peak current-voltage (I-V) curves were built from current traces obtained with a series of step depolarizations associated with a P/4 protocol for on-line leak and capacitive current subtraction. Peak currents were transformed into peak current densities by dividing them by the membrane capacitance of each cell. Transient potassium current (I_A_) was isolated by using voltage protocols, taking advantage of the distinct voltage-dependent inactivation of the underlying potassium channels [22]. First, total voltage-activated potassium current was measured in cells held at −80 mV, in which a 1 s conditioning pulse to −100 mV was delivered prior to 250 ms step depolarizations, ranging from −20 to +20 mV in 10 mV increments. Then, a depolarizing 1 s conditioning pulse to −20 mV was applied, sufficient to inactivate I_A_, such that the outward current evoked by subsequent step depolarizations was mostly comprised of a delayed rectifying potassium current. I_A_ was finally revealed by subtracting the delayed rectifying current from total current. Peak amplitude of I_A_ was used to determine percent block by IQM-PC332. I_A_ inactivation was fitted to a biexponential process with an equation of the form y = y_0_ + A_f_ exp − (x − x_0_)/τ_f_ + A_s_ exp − (x − x_0_)/τ_s_, where τ_f_ and τ_s_ represent the fast and slow time constants, respectively, and A_f_ and A_s_ represent the amplitudes of the corresponding kinetic components.

Current trace analysis, curve fitting, and data presentation were performed with Igor Pro 5.0 (Wavemetrics Inc.), Fitmaster (HEKA Electronic, Lambrecht, Germany) and GraphPad Prism 8 (GraphPad Software, La Jolla, CA, USA).

### 4.5. Statistics

Data are given as the mean ± standard error of the mean (SEM) of the corresponding number of behavioural measurements or cells used. All collected data were first tested for normality as well as for homogeneity of variances. In behavioural experiments, differences between groups were assessed by two-way analysis of variance (ANOVA) for repeated measurements followed by a Bonferroni’s test for multiple comparisons. Two-way ANOVA and paired or unpaired Student’s *t*-tests were used for data comparisons in electrophysiological experiments. Post hoc tests were run only if F achieved *p* < 0.05, and the threshold for statistical significance was *p* < 0.05 throughout. GraphPad Prism 8 (GraphPad Software, La Jolla, CA, USA) was employed for these analyses. Differences with *p* < 0.05 were considered significant.

## Data Availability

Not applicable.

## Supplementary Materials

The following supporting information can be downloaded at: https://www.mdpi.com/article/10.3390/ijms23042142/s1.

## Abbreviations

BDNF	Brain derived neurotrophic factor
Ca_v_	Voltage-activated calcium channel/current
CCI	Chronic constriction injury
DMEM	Dulbecco’s modified Eagle’s Medium-low glucose
DREAM	Downstream regulatory element antagonist modulator
DRG	Dorsal root ganglia
ED_50_	Effective dose 50%
HVA	High voltage-activated
IC_50_	Inhibitory concentration 50%
i.p.	Intraperitoneal
i.pl.	Intraplantar
IQM-PC332	(2-[2-(3,4-Dichlorophenyl)acetylamino]-4-(4′-n-butylphenyl)benzoic acid)
KChIP3	Potassium channel interacting protein 3
K_D_	Dissociation constant
K_v_	Voltage-activated potassium channel/current
MPE	Maximum possible effect
Na_v_	Voltage-activated sodium channel/current
PWT	Paw withdrawal threshold
TRPV1	Transient receptor potential vanilloid-1
V_h_	Holding voltage
V_comm_	Voltage command
V_r_	Resting membrane potential
