# Supplementary material for: IQM-PC332, a Novel DREAM Ligand with Antinociceptive Effect on Peripheral Nerve Injury-Induced Pain
Source: Int J Mol Sci. 2022 Feb 15;23(4):2142. doi: 10.3390/ijms23042142 (PMC8876042; doi:10.3390/ijms23042142)
Supplement: Supplementary file 1 [file ijms-23-02142-s001.zip › ijms-1544680-supplementary.pdf]

International Journal of *Molecular Sciences*

MDPI

Article

IQM-PC332, a Novel DREAM Ligand with Antinociceptive Effect on Peripheral Nerve Injury-Induced Pain

Paula G. Socuéllamos [1,2,†], Luis A. Olivos-Oré [1,3,†], María Victoria Barahona [1,3,†], Pilar Cercós [4], Marta Pérez Pascual [1], Marina Arribas-Blázquez [1,3], José Ramón Naranjo [5,6], Carmen Valenzuela [2,7], Marta Gutiérrez-Rodríguez [4,*] and Antonio R. Artalejo [1,3,*]

[1] Department of Pharmacology and Toxicology, Veterinary Faculty, and Instituto Universitario de Investigación en Neuroquímica (IUIN), Universidad Complutense de Madrid, 28040 Madrid, Spain; olivos@ucm.es (L.A.O.-O.); vbg@ucm.es (M.V.B.); martpe13@ucm.es (M.P.P.); marina.arribas@vet.ucm.es (M.A.-B.); artalejo@ucm.es (A.R.A.)
[2] Instituto de Investigaciones Biomédicas Alberto Sols (IIBM), CSIC-UAM, 28029 Madrid, Spain; paulagarcia@iib.uam.es (P.G.S.); cvalenzuela@iib.uam.es (C.V.)
[3] Instituto de Investigación Sanitaria San Carlos, 28040 Madrid, Spain
[4] Instituto de Química Médica (IQM-CSIC), 28006 Madrid, Spain; pcercos@nebrija.es
[5] Centro Nacional de Biotecnología–Consejo Superior de Investigaciones Científicas (CSIC), 28049 Madrid, Spain; naranjo@cnb.csic.es
[6] Centro de Investigación Biomédica en Red Enfermedades Neurodegenerativas (CIBERNED), 28031 Madrid, Spain
[7] Centro de Investigación Biomédica en Red Enfermedades Cardiovasculares (CIBERCV), 28029 Madrid, Spain
* Correspondence: artalejo@ucm.es (A.R.A.); mgutierrez@iqm.csic.es (M.G.-R.)
Tel.: +34-91-258-7493 (M.G.-R.); +34-913-943-851 (A.R.A.)
† These authors contributed equally to this work.

Citation: Socuéllamos, P.G.; Olivos-Oré, L.A.; Barahona, M.V.; Cercós, P.; Pascual, M.P.; Arribas-Blázquez, M.; Naranjo, J.R.; Valenzuela, C.; Gutiérrez-Rodríguez, M.; Artalejo, A.R. IQM-PC332, a Novel DREAM Ligand with Antinociceptive Effect on Peripheral Nerve Injury-Induced Pain. *Int. J. Mol. Sci.* **2022**, *23*, 2142. https://doi.org/10.3390/ijms23042142

Academic Editor: Irmgard Tegeder

Received: 22 December 2021
Accepted: 12 February 2022
Published: 15 February 2022

Publisher's Note: MDPI stays neutral with regard to jurisdictional claims in published maps and institutional affiliations.

Copyright: © 2022 by the authors. Licensee MDPI, Basel, Switzerland. This article is an open access article distributed under the terms and conditions of the Creative Commons Attribution (CC BY) license (https://creativecommons.org/licenses/by/4.0/).

Supplementary Materials:

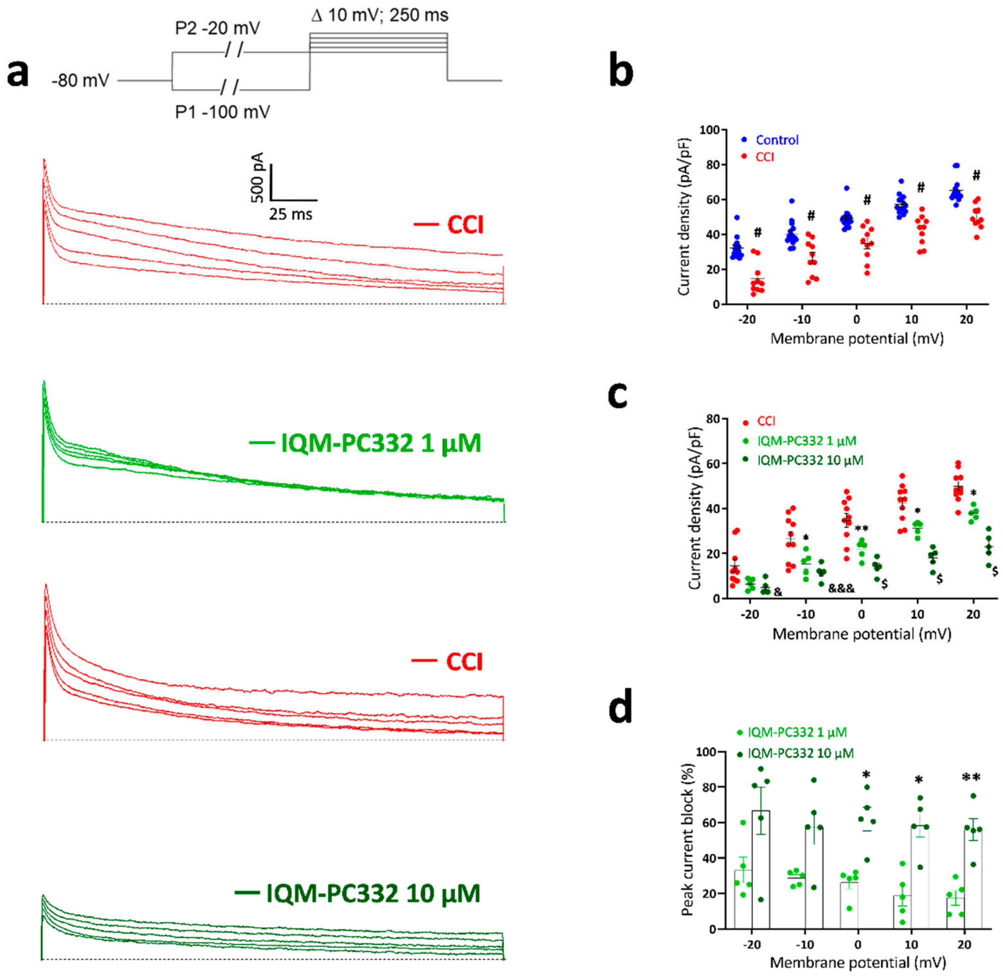

Figure S1. Effect of IQM-PC332 on I_A currents of DRG neurons from CCI animals. **a)** Representative recordings of I_A isolated by using the voltage protocol depicted on top of the panels. The effect of IQM-PC332 1 µM (*upper panel*) or 10 µM (*lower panel*) is shown. **b)** Peak current density to voltage (I-V) relation in Control (membrane capacitance of 32.23 ± 0.94 pF; n = 14 cells) and CCI (membrane capacitance of 31.40 ± 0.93 pF; n = 10 cells) DRG neurons. **c)** I-V relation in the absence (CCI) and presence of IQM-PC332 1 µM (membrane capacitance of 32.62 ± 1.23 pF; n = 5 cells) and 10 µM (membrane capacitance of 30.19 ± 1.30 pF; n = 5 cells). **d)** Percent block of peak I_A by IQM-PC332 (1 µM and 10 µM) at different potentials. Data are expressed as mean ± SEM. Statistical significance was assessed by two-way ANOVA, followed by a Bonferroni's test for multiple comparisons with respect to Control (**b**), CCI (**c**) and IQM-PC332 1 µM (**d**). * and &: p < 0.05; **: p < 0.01; &&&: p < 0.001; # and $: p < 0.0001.

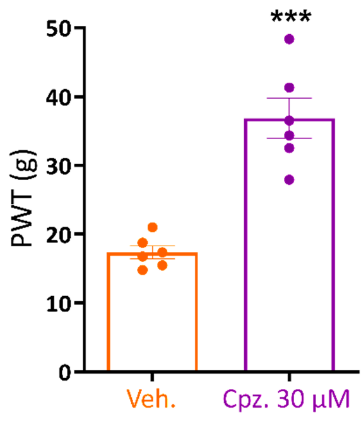

Figure S2. Effect of capsazepine on mechanical sensitivity in CCI animals. Paw withdrawal threshold (PWT) as an indicator of mechanical sensitivity was determined after i.pl. administration of 30 μg capsazepine (Cpz.), a TRPV1 channel blocker, or vehicle (Veh.; Tween 80/ethanol/saline; 10/10/80). Data are expressed as the mean ± SEM of 6 measurements (n = 6 animals). Statistical significance was assessed by using Student's *t*-test for paired comparisons. ***, $P < 0.001$ with respect to Veh.
